# Helper T Cell Epitope-Mapping Reveals MHC-Peptide Binding Affinities That Correlate with T Helper Cell Responses to Pneumococcal Surface Protein A

**DOI:** 10.1371/journal.pone.0009432

**Published:** 2010-02-25

**Authors:** Rajesh Singh, Shailesh Singh, Praveen K. Sharma, Udai P. Singh, David E. Briles, Susan K. Hollingshead, James W. Lillard

**Affiliations:** 1 Department of Microbiology, Biochemistry, and Immunology, Morehouse School of Medicine, Atlanta, Georgia, United States of America; 2 Department of Microbiology and Immunology, University of Louisville School of Medicine, Louisville, Kentucky, United States of America; 3 Department of Pathology, Microbiology and Immunology, University of South Carolina School of Medicine, Columbia, South Carolina, United States of America; 4 Department of Microbiology, University of Alabama at Birmingham School of Medicine, Birmingham, Alabama, United States of America; University of Toronto, Canada

## Abstract

Understanding the requirements for protection against pneumococcal carriage and pneumonia will greatly benefit efforts in controlling these diseases. Several proteins and polysaccharide capsule have recently been implicated in the virulence of and protective immunity against *Streptococcus pneumonia*. Pneumococcal surface protein A (PspA) is highly conserved among *S. pneumonia* strains, inhibits complement activation, binds lactoferrin, elicits protective systemic immunity against pneumococcal infection, and is necessary for full pneumococcal virulence. Identification of PspA peptides that optimally bind human leukocyte antigen (HLA) would greatly contribute to global vaccine efforts, but this is hindered by the multitude of HLA polymorphisms. Here, we have used an experimental data set of 54 PspA peptides and *in silico* methods to predict peptide binding to HLA and murine major histocompatibility complex (MHC) class II. We also characterized spleen- and cervical lymph node (CLN)-derived helper T lymphocyte (HTL) cytokine responses to these peptides after *S. pneumonia* strain EF3030-challenge in mice. Individual, yet overlapping peptides, 15 amino acids in length revealed residues 199 to 246 of PspA (PspA_199–246_) consistently caused the greatest IFN-γ, IL-2, IL-5 and proliferation as well as moderate IL-10 and IL-4 responses by *ex vivo* stimulated splenic and CLN CD4^+^ T cells isolated from *S. pneumonia* strain EF3030-challeged F_1_ (B6×BALB/c) mice. IEDB, RANKPEP, SVMHC, MHCPred, and SYFPEITHI *in silico* analysis tools revealed peptides in PspA_199–246_ also interact with a broad range of HLA-DR, -DQ, and -DP allelles. These data suggest that predicted MHC class II-peptide binding affinities do not always correlate with T helper (Th) cytokine or proliferative responses to PspA peptides, but when used together with *in vivo* validation can be a useful tool to choose candidate pneumococcal HTL epitopes.

## Introduction

Pneumococcal pneumonia is the most common cause of childhood deaths in the developing world and among the top ten causes of death in aged populations worldwide; recently, antibiotic-resistant *S. pneumonia* strains have emerged [1], [2], [3], [4]. Hence, vaccines against these strains are greatly needed. This study characterizes the HTL epitopes of a candidate pneumococcal vaccine antigen, PspA, which is a highly conserved, cell wall-associated surface protein that plays a major role in pneumococcal virulence by binding human lactoferrin and interferes with complement deposition on the bacterial surface [5]. During the course of invasive disease, antibodies against PspA peak during the convalescent phase, but CD4^+^ T cell help is required for optimal protective immune responses to PspA [6], [7].

A central event in the adaptive immune response to invasive microorganisms is the specific recognition of processed antigens bound to the peptide-binding region of MHC class II molecules on the surface of antigen-presenting cells. These peptide antigens are subsequently detected by the T cell receptor (TCR) of CD4^+^ T cells, which proliferate, secrete cytokines, and differentiate into antigen-specific Th effector cells. To induce protective immunity, HTL epitopes contained in synthetic peptide vaccines must: (i) match those naturally presented to the immune system during infection, (ii) be recognized by the majority of the human population, and (iii) induce an appropriate effector immune response to eliminate the pathogen of interest. Single epitope-based vaccines may, however, have drawbacks. For example, the mono-specificity of the induced immune response might miss the emergence of sequence mutants that would potentially escape the vaccine's protective effect [8]. It is also unlikely that T cells from genetically distinct populations would recognize, and respond to a single peptide epitope.

These obstacles are secondary to the wide-ranging polymorphisms of HLA molecules that present antigenic peptides to T cells. Indeed, a unique set of epitopes from a given protein antigen will be presented to T cells of an individual bearing hundreds of unique HLA molecules. Additionally, some HLA molecules may not be able to bind to any of the peptides derived from a given protein [9], [10]. The major challenge of peptide-based vaccines is the identification of one or more epitope(s) that bind to many HLA alleles and cover close to 100% of the genetically diverse human population [11]. Thus, the identification of peptides that bind to multiple HLA types, the so-called “promiscuous” or “universal” epitope(s), could lead to effective coverage of the human population using peptide-based vaccine.

Until recently, the search for immunodominant peptides relied on the direct testing of overlapping peptides or peptide libraries. Fortunately, the identification of MHC binding motifs allowed for the prediction of potential T cell epitopes [12], [13]. To identify the immunodominant epitopes of PspA, we used *in silico* MHC affinity measurement methods using both affinity data from the Immune Epitope Database and Analysis Resource (IEDB) [14], eluted peptide data from the SYFPEITHI [12] database as well as RANKPEP [15], SVMHC [16], and MHCPred tools [17], [18], which predicted the PspA peptides that bind HLA-DR, -DQ, and -DP alleles. To correlate these predictions with *in vivo* immunogenicity, PspA-specific HTL proliferation and cytokine responses were measured and correlated with predicted peptide-MHC binding affinities. A novel human isolate of capsular group 19 pneumococci, which was passed in mice to yield *S. pneumonia* strain EF3030, which has a greater propensity to cause nasal or pulmonary infections than sepsis when given intranasally, was used to accomplish this objective [19]. Further, F1 (B6×Balb/c) mice have reduced susceptibility to *S. pneumonia* strain EF3030 and express functional I-A^b^, I-A^d^, I-E^b^, and I-E^d^. After these mice were nasally challenged with *S. pneumonia* strain EF3030, CLN- and spleen-derived CD4^+^ T cells were isolated and *ex vivo* stimulated with PspA peptides. Together, these *in silico* and *in vivo* methods revealed immunodominant PspA HTL epitopes that might serve as vaccine antigens.

## Results

### Peptide Selection, Binding Analysis, and Overview of PspA Predicted Secondary Structure

The aligned PspA amino acid sequence using 24 unrelated *S. pneumonia* strains, was previously shown to contain helical and charged immunogenic domains (i.e., Regions A, A*, B, and C) [20]. As reported previously, the secretion signal peptide for PspA extends into the first 50 amino acids and has >50% amino acid identity among strains. Region A encodes the first 100 amino acids (∼300 nucleotides) of PspA, beginning with the first amino acid of the mature protein. PspA is less conserved over the second half of Region A, where sequences begin to diverge and fall into groups. The amino terminal end of Region A* is hypervariable, but the C-terminal end of Region A* and much of Region B are more conserved among strains. Region C is proline-rich.

**Figure 1:**
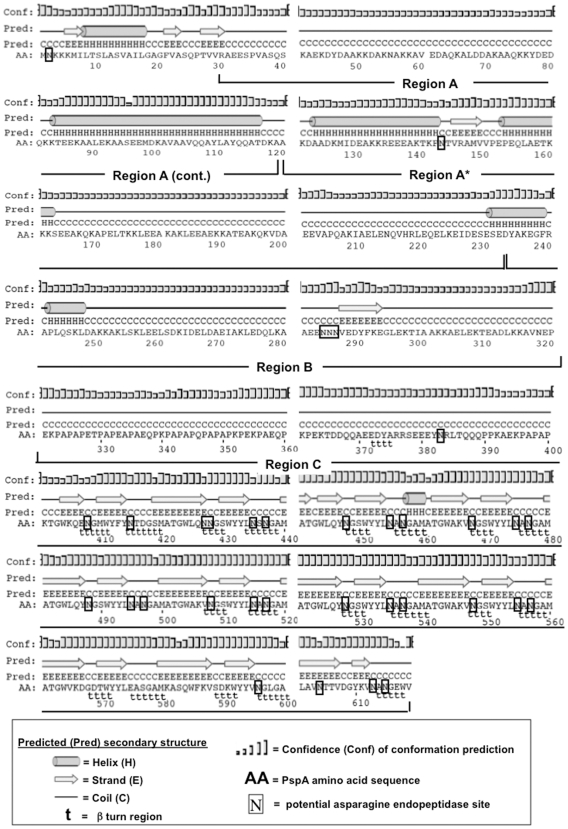
Modular PspA amino acid sequence showing regions of predicted immunogenicity and secondary structure. Major domains of PspA are indicated. The aligned amino acid sequence shows the previously defined PspA windows A, A*, B and C. The PspA amino acid (AA) sequence was used to predict helical (H), coiled (C), α strand (E), β turns (t), and asparagine endopeptidase sites (N).

We created individual, yet overlapping peptides, that were 15 amino acids in length (Table 1). The entire sequence of PspA was used to predict the protein structure as well as β turn (t) using PSIPRED (http://bioinf.cs.ucl.ac.uk/psipred/) [21] and COUDES (http://bioserv.rpbs.jussieu.fr/Coudes/index.html) [22] methods. Coiled–coiled (C) as well as helical (H) structures were noted throughout PspA (Figure 1). There were no β turns or potential asparagine (N) endopeptidase sites in Regions A or A*.The majority of Region B is coiled with small helix (PspA_242–246_) and strand (PspA_286–293_) domains. In contrast, Region C displays an array of complex secondary structures as well as numerous potential N endopeptidase sites. The latter sites are typically found in bacterial cell wall-associated domains and known to enhance antigen-processing for MHC presentation [23].

**Table 1:** Overlapping PspA peptides and antigenic region description.

Peptide	Antigenic epitope region	Peptide	Antigenic epitope region
01-MNKKKMILTSLASVA	Leader	28-TIAAKKAELEKTEAD	Region B
02-ASVAILGAGFVASQP	Leader	29-TEADLKKAVNEPEKP	Region B
03-ASQPTVVRAEESPVA	Leader/Region A	30-PEKPAPAPETPAPEA	Region B/C
04-SPVASQSKAEKDYDA	Region A	31-APEAPAEQPKPAPAP	Region C
05-DYDAAKKDAKNAKKA	Region A	32-APAPQPAPAPKPEKP	Region C
06-AKKAVEDAQKALDDA	Region A	33-PEKPAEQPKPEKTDD	Region C
07-LDDAKAAQKKYDEDQ	Region A	34-KTDDQQAEEDYARRS	Region C
08-DEDQKKTEEKAALEK	Region A	35-ARRSEEEYNRLTQQQ	Region C
09-ALEKAASEEMDKAVA	Region A	36-TQQQPPKAEKPAPAP	Region C
10-KAVAAVQQAYLAYQQ	Region A	37-APAPKTGWKQENGMW	Region C
11-AYQQATDKAAKDAAD	Region A	38-NGMWYFYNTDGSMAT	Region C
12-DAADKMIDEAKKREE	Region A*	39-SMATGWLQNNGSWYY	Region C
13-KREEEAKTKFNTVRA	Region A*	40-SWYYLNSNGAMATGW	Region C
14-TVRAMVVPEPEQLAE	Region A*	41-ATGWLQYNGSWYYLN	Region C
15-QLAETKKKSEEAKQK	Region A*	42-YYLNANGAMATGWAK	Region C
16-AKQKAPELTKKLEEA	Region A*	43-GWAKVNGSWYYLNAN	Region C
17-LEEAKAKLEEAEKKA	Region A*	44-LNANGAMATGWLQYN	Region C
18-EKKATEAKQKVDAEE	Region A*	45-LQYNGSWYYLNANGA	Region C
19-DAEEVAPQAKIAELE	Region A*	46-ANGAMATGWAKVNGS	Region C
20-AELENQVHRLEQELK	Region A*	47-VNGSWYYLNANGAMA	Region C
21-QELKEIDESESEDYA	Region A*/B	48-GAMATGWLQYNGSWY	Region C
22-EDYAKEGFRAPLQSK	Region B	49-GSWYYLNANGAMATG	Region C
23-LQSKLDAKKAKLSKL	Region B	50-MATGWAKVNGSWYYL	Region C
24-LSKLEELSDKIDELD	Region B	51-WYYLNANGAMATGWV	Region C
25-DELDAEIAKLEDQLK	Region B	52-TGWVKDGDTWYYLEA	Region C
26-DQLKAAEENNNVEDY	Region B	53-YLEASGAMKASQWFK	Region C
27-VEDYFKEGLEKTIAA	Region B	54-QWFKVSDKWYYVNGL	Region C

Individual, yet overlapping, *Streptococcus pneumonia* strain R6 PspA peptides, 15 amino acids in length were used in *ex vivo* and *in silico* assays. The antigenic epitope regions based on homologous alignment of PspA amino acid sequences from other strains were previously described as leader, A, A*, B, and C regions [20].

Next, the PspA peptide dataset was was used to determine MHC II binding affinities (Table 2). These data span a total of 16 human and 4 mouse MHC class II types. IEDB, MHCPred, RANKPEP, SVMHC and SYFPEITHI MHC class II epitope databases scanned the entire sequence of PspA. In brief, PspA peptides were compared with archived peptide datasets of previously measured peptide-MHC class II affinities. Peptides were classified into binders (IC_50_<500 nM) and non-binders (IC_50_≥500 nM) based on *in silico*-derived binding affinities. This analysis revealed that nearly all PspA peptides could potentially bind a variety of mouse and human MHC class II molecules. Finally, the amino acid sequence comprising PspA peptides 19 to 22 (or PspA_199–246_) was aligned with sequences from nearly 100 clinically relevant family 1 *S. pneumonia* strains (Table 3). PspA_199–246_ is highly conserved among *S. pneumonia* strains and contains the C-terminal end of Region A* and the beginning of Region B [20].

**Table 2:** Overview of PspA peptide predicted binding affinities to MHC class II alleles.

PspA Peptide Number	IC_50_ *in silico* prediction (nM)				
	I-A^b^	I-A^d^	I-E^b^	I-E^d^	HLA-DRB, -DP, -DQ Alleles with IC_50_<500 nM predicted peptide-binding affinity
01	312	177	–	–	1*0101, 1*0301, 1*0401, 1*0404, 1*0405, 1*0701, 1*0802, 1*0901, 1*1101, 1*1302, 4*0101, 5*0101
02	316	81	–	372	1*0101, 1*0401, 1*0404, 1*0701, 1*1501,DP4, DPw4, DQ1, DQ5
03	171	21	358	–	1*0101, 1*0301, 1*0401, 1*0901, 1*0701, 5*0101
04	71	302	–	–	1*0101, 1*0404, 1*0701, DP4, DP9
05	–	–	–	–	1*0101, 1*0404, 1*0701, 1*0802, 5*0101, DQ5
06	167	56	–	–	1*0101, 1*0404, 1*0701, DP9, DQ1, DQ5
07	338	–	–	–	1*0101, 1*0404, 1*0701, DQ8
08	118	17	–	–	1*0101, 1*0404, 1*0701
09	219	57	499	–	1*0101, 1*0404, 1*0701, DP4, DPw4,
10	193	12	–	337	1*0101, 1*0404, 1*0701, 4*0101, DQ1, DQ2, DQ5, DQ7
11	223	215	–	–	1*0101, 1*0401,1*0701, 1*0901, 3*0101, DQ8
12	380	488	–	–	1*0101, 1*0301, 1*0701, 5*0101, DQ2
13	241	–	348	–	1*0101, 1*0401,1*0701, 1*0802, 5*0101, DP9, DPw4, DQ7, DQ8
14	210	34	–	–	1*0101, 1*0401, 1*0701, 1*0405, 5*0101
15	354	46	–	–	1*0101, 1*0401,1*0701, 1*0901, 5*0101, DP9 , DQ1, DQ2
16	182	99	–	344	1*0101, 1*0401,1*0701, DP9 , DQ7
17	49	–	–	–	1*0101, 1*0401,1*0701, DP9, DQ2
18	208	12	–	307	1*0101, 1*0401,1*0701, 1*0901, DQ8
19	205	104	–	–	1*0101, 1*0401,1*0701, DQ2
20	485	–	493	–	1*0101, 1*0301,1*0401,1*0701, DP4, DP9, DPw4, DQ1
21	180	–	–	–	1*0101
22	121	42	–	–	1*0101, 1*0401,1*0701,DP4, DP9, DQ1, DQ5
23	452	–	412	–	1*0101, 1*0401, 1*0405, 1*0701, 1*0802, 1*0901, 5*0101,DP4, DP9
24	374	–	–	–	1*0101, 1*0401,1*0701,
25	365	461	–	–	1*0101, 1*0401,1*0701, DP9, DQ2
26	462	6	–	–	1*0101, 1*0401,1*0405,1*0701, 5*0101, DQ2
27	249	112	–	–	1*0101,1*0401, 1*0405, 1*0701, 1*0901, 5*0101,DP4, DP9, DQ5
28	124	190	–	–	1*0101, 1*0401,1*0701, DP9, DQ7, DQ8
29	97	10	411	–	1*0101, 1*0401,1*0701, 1*0901, 5*0101, DQ5
30	99	125	–	–	1*0101, 1*0401,1*0701, DQ2
31	173	57	–	–	1*0101, 1*0401,1*0701, DQ2
32	350	15	–	–	1*0101, 1*0401,1*0701, DQ2
33	239	–	–	–	1*0101, 1*0401,1*0701,
34	349	28	–	–	1*0101, 1*0401,1*0701, DQ1
35	457	155	210	–	1*0101, 1*0401,1*0701, DPw4
36	211	65	–	479	1*0101, 1*0401,1*0701, DQ2
37	369	20	–	381	1*0101, 1*0401,1*0701,
38	140	105	–	378	1*0101, 1*0401, 1*0405, 1*0701, 1*1101, DQ1, DQ5, DQ7, DQ8
39	204	121	–	285	1*0401, 1*0405, 1*0701, 1*1302, 1*1501
40	348	76	381	–	1*0101, 1*0401, 1*0404, 1*0405, 1*0701, 1*0901, 1*1101, 1*1302, 1*1501, 5*0101,DP4, DPw4
41	264	60	268	–	1*0101,1*0401, 1*0404, 1*0405, 1*0701, 1*1302, 1*1501
42	256	175	–	357	1*0101, 1*0401, 1*0404, 1*0405, 1*0701, 1*0901, 1*1101, 1*1302, DQ8
43	329	21	–	–	1*0101, 1*0401,1*0405, 1*0701, 1*1501, 3*0101, 5*0101
44	274	7	–	–	1*0101, 1*0401, 1*0405, 1*0701, 1*1101, 1*1302, DQ7
45	485	60	–	476	1*0101, 1*0401, 1*0404, 1*0405, 1*0901, 1*1101, 3*0101, 5*0101
46	–	44	–	–	1*0101, 1*0401, 1*0405, 1*0701, 1*1101
47	255	21	–	–	1*0101, 1*0401, 1*0404, 1*0405, 1*0701, 1*0901, 1*1101, 1*1302, 3*0101, 5*0101
48	264	47	–	–	1*0101,1*0401, 1*0404, 1*0405, 1*0701, 1*1302, 1*1501
49	255	120	–	–	1*0101, 1*0401, 1*0404, 1*0405, 1*0701, 1*0901, 1*1101, 1*1302, 5*0101
50	329	–	–	–	1*0101, , 1*0401, 1*0405, 1*0701, 1*1501
51	255	45	–	–	1*0101, 1*0401, 1*0404, 1*0405, 1*0701, 1*0901, 1*1101, 1*1302, 5*0101,DP4, DQ5
52	392	204	–	–	1*0101, 1*0301,1*0401, 1*1501,1*0701
53	80	27	–	–	1*0101, 1*0401, 1*0701, 1*0901, 5*0101,DP4, DPw4, DQ5
54	387	234	–	170	1*0101, 1*0401, 1*0701, 1*1501, 3*0101, DQ8

*Dashes (–) represent the predicted affinity of peptides that poorly (i.e., IC_50_>500 nM) bind mouse I-A^b^, I-E^b^, I-A^d^, or I-E^d^ alleles. Similarly, absent HLA alleles are those that poorly (i.e., IC_50_>500 nM) bind the corresponding peptide.

**Table 3:** Alignment of PspA_199–256_ amino acid sequences from family 1 Pneumococci strains.

Strain	NCBI Accession Number	*Conserved amino acid sequence
D39/R6	NP_357715	DAEEVAPQAKIAELENQVHRLEQELKEIDESESEDYAKEGFRAPLQSK
WU2	AAF27710	EVAPQAKIAELENQVHRLEQELKEIDESESEDYAKEGFRAPLQSK
195	AAF68105	EEVAPQAKIAELENQVHRLEQELKEIDESDSEDYIKEGFRAPLQSE
SP19	AAF68093	AEEVAPQAKIAELENQVHKLEQKLKEIDESDSEDYVKEGFRAPLQSE
CGSP14, R41	YP_001834837, ABY67182	EVAPQAKIAELENQVHRLEQDLKDINESDSEDYVKEGLRAPLQSE
RHG79, OVP-41721	ABY67197, ACR50702	HAEEVAPQVKIAELENQVHKLEQKLKEIDESDSEDYVKEGLRAPLQSE
EF3030	to be determined	.. EVALQAKIAELENQVHRLETELKEIDESDSEDYVKEGLRVPLQSE
c2, OVP-43533, OVP-42723, OVP-43431, R24729, DBL5, HUB-6893, St 371/00	ACM45238, ACR50689, ACR50693, ACR50694, ABY67184, AAF27706, ACR50678, ABR53733	HAKEVAPQAKIAELENQVHRLEQDLKDINESDSEDYVKEGLRAPLQSE
L81905, RH5, BG9739, MC-247	AAF27705, ABV60383, AAF27700, ACR50682	RAKEVVLQAKIAELENEVHKLEQKLKEIDESDSEDYVKEGFRAPLQSE
70585	YP_002739507	RAKEVALQAKIAELENEVHRLETKLKEIDESDSEDYVKEGLRAPLQSE
AC94	AAF27698	RAKEVALQAKIAELENEVHRLETELKEIDESDSEDYVKEGLRVPLQSE
SP6-BS73, EF6796, BG9163, RHG63	ZP_01819322, AAF27709, AAF27711, ABY67195	EVALQAKIAELEYEVQRLEKELEEINESDSEDYAKEGFRAPLQSK
SP18-BS74	ZP_01829602	HAEEVVPQAKIAELENEVQKLEKDLKEIDESDSEDYVKEGLRAPLQSE
SP200, MC-332, SP221	AAF67354, ACR50683, AAF68099	RAKEVALQAKIAELENQVHRLETELKEIDESDSEDYVKEGLRVPLQSE
BG8838, R30318	AAF27703, ABW07806	HAEEVVPQAKIAELENEVQKLEKDLKEIDESDSEDYVKEGLRAPLQSE
R30397, R171, BG6692	ABV60382, ACH72677, AAF27704	HAEEVVPQAKIAELENEVQKLEKDLKEIDESDSEDYVKEGLRAPLQSE
HUB-7682	ACR50697	RAKEVALQAKIAELENEVHRLETKLKEIDESDSEDYVKEGLRAPLQSE
130	AAF68103	HAEEVVPQAKIAELENEVQKLEKDLKEIDESASEDYVKEGLRAPLQSE
R30318	ABW07807	RAKEVALQAKIAELENEVHRLETKLKETDESDSEDYVKEGLRAPLQSE
OVI-2328	ACR50701	HAKEVVPQAKIAELENEVQKLEKDLKEIDESDSEDYVKEGLRAPLQSE
*CDC1873-00, ST858, *SP6-BS73, *EF6796, ST860, *SRF10, SP23-BS72, *g5, E134, BG9163	ZP_02709307†, ABN71686, ZP_01820249†, AAD00184†, ABN71687, AAF73809†, ZP_01835080, AAF73801†, AAF13457, AAF13460	DAEEYALEAKIAELEYEVQRLEKELKEIDESDSEDYLKEGLRAPLQSK
232	AAF68104	HAEEVVPQAKIAELENEVQKLEKDLKEIDESASEDYVKEGLRAPLQSE
P1031, R30087	YP_002737416, ABY67187	RAKEVALQAKIAELENEVHRLETKLKETDESDSEDYVKEGLRAPLQSE
CDC3059-06	ZP_02717970	HAEEVAPQAKIAELEHEVQKLEKALKEIGESDSEDYVKEGLRAPLQSE
OVP-42725	ACR50703	LFLQAKIAELENEVHKLEQKLKEIDESDSEDYVKEGFRAPLQSE
PN124	AAN37735	AKIAELENQVHRLEQDLKDINESDSEDYVKEGFRAPLQSE
DBL6A	AAF27701	RAKEVVLQAQIAELENEVHKLEPKLKEIDESDSEDYVKEGFRAPLQSE
St 435/96	AAL92492	HAEEVAPQAKIAELEHEVQKLEKALKEIDESDSEDYVKEGLRAPLQFE
EF10197	AAF27708	RAKEVVLHAKLAELENEVHKLDQKLKEIDESDSEDYVKEGFRAPLQSE
R402	ABY67181	HAEEVAPQAKIAELEHEVQKLEKALKEIDESDSEDYVKEGLRAPLQFE
DBL1	AAF27702	RAKEVALQAKIAELENEVYRLETELKGIDESDSEDYVKEGLRAPLQSE
HUB-4197, 237	ACR50680, AAF68102	HAEEVAPQAKIAELEHEVQKLEKALKEIDESDSEDYVKEGLRAPLQFE
c1, SP194, RHG95, HUB-2371, PC4, RH21, RH12	ACM45237, AAF68092, ABV60384, ACR50685, ABV30914, ABY67192, ABW07810	RAKEVALQAKIAELENEVYRLETELKGIDESDSEDYVKEGLRAPLQSE
SP23-BS72, SP196, URSP2, 233, 152, 164, BG8743, 183, HUB-6892, 90, 177, 137, 39, RH9	ZP_01834257, AAF67355, AAR20918, AAF70097, AAF70096, AAF70094, AAF27699, AAF70095, ACR50684, AAF70093, AAF70091, AAF70090, AAF70092, ABW07809	KYALEAKIAELEYEVQGLEKELKEIDESDSEDYIKEGLRAPLQSK
R23661, R30360, OVP-40742	ABV30913, ABY67189,	KYALEAKIAELEYEVQRLEKEIKEIDESDSEDYLKEGLRAPLQSE
R11561	ACH72679	EVAPQAKIAELENQVHRLEQDL-----SDSEGYVKEGLRAPLQSE
E134	AAF27707	KYALEAKISELEYEVQGLGKELKEIDESDSEDYXKEGLRAPLQSK
SP356	AAN37734	IAELENEVYRLETELKGIDESDSEDYVKEGLRAPLQSE
R83	ACH72676	KYALEAKIAELEYEVQRVEKEIK--DESDSEDYLKEGLRAPLQSE
P105	ABE67219	LEKEIKEIDESDSEDYLKEGLRAPLQSE
P755, P13	ABE67236, ABE67218	LKEIDESDSEDYVKEGFRAPLQSE
P1151	ABE67224	LKEIDESDSEDYIKEGVRAPLQSK
P308	ABE67222	LKEIDESDSEDYIKEGLRAPLQSK
P176, 371/00, P1161	ABE67232, AAL92493, ABE67225	LKEIDESDSEDYVKEGLRAPLQSE

*Alignment of conserved *Streptococcus pneumonia* PspA_199–256_ amino acid sequences appear as white text in black boxes.

†NCBI accession number of PspC that align with PspA_199–246_.

### PspA Peptide-Specific Systemic and Mucosal CD4^+^ T Cell Proliferation Responses

**Figure 2:**
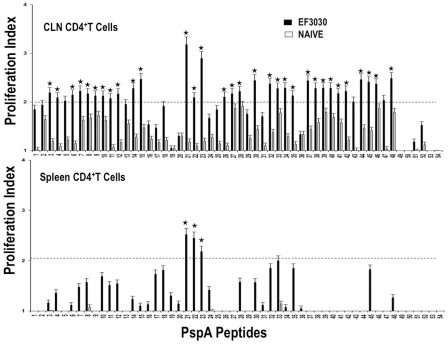
Proliferation responses of PspA peptide-specific systemic and mucosal CD4^+^ T cells during pneumococcal carriage. Spleen and cervical lymph node (CLN) lymphocytes were isolated from F1 (B6×Balb/c) mice, 28 days after intranasal challenge with *Streptococcus pneumonia* strain EF3030 (▪) and naïve (□). CD4^+^ T cells were incubated with 1 µM of PspA peptide (15 amino acid peptides that overlapped every 11 residues) plus mitomycin C-treated naïve syngeneic feeder cells, for 3 days, at a ratio of 5∶1×10^6^ cells. Proliferation was measured by BrdU incorporation, which was measured by ELISA. The data presented are the mean OD_450_. Experimental groups consisted of 10 mice. The results were expressed as the mean ± the standard error mean (SEM) of the response from 3 replicate determinations of three independent experiments.

To better determine whether predicted PspA peptide-MHC class II binding affinities corresponded with HTL proliferation, PspA peptide-specific CD4^+^ T cell responses were characterized 28 days after *S. pneumoniae* strain EF3030 or mock (naïve) challenge. PspA peptide-specific proliferative responses by naïve CD4^+^ T cells were relatively low (Figure 2). However, spleen- or CLN-derived CD4^+^ T cell from *S. pneumonia* strain EF3030-challenged mice showed selective yet significant proliferation indexes to PspA peptides. Spleen-derived HTLs from *S. pneumonia* strain EF3030-challenged mice significantly proliferated in response to PspA peptides 21, 22, and 23 than compared to naïve controls. CLN CD4^+^ T cell PspA peptide-specific proliferation responses were moderately higher than similar cells isolated from the spleen of *S. pneumonia* strain EF3030-challenged mice, with comparatively higher responses to PspA peptides 21 and 23.

### PspA Peptide-Specific T Helper Cytokine Profiles

**Figure 3:**
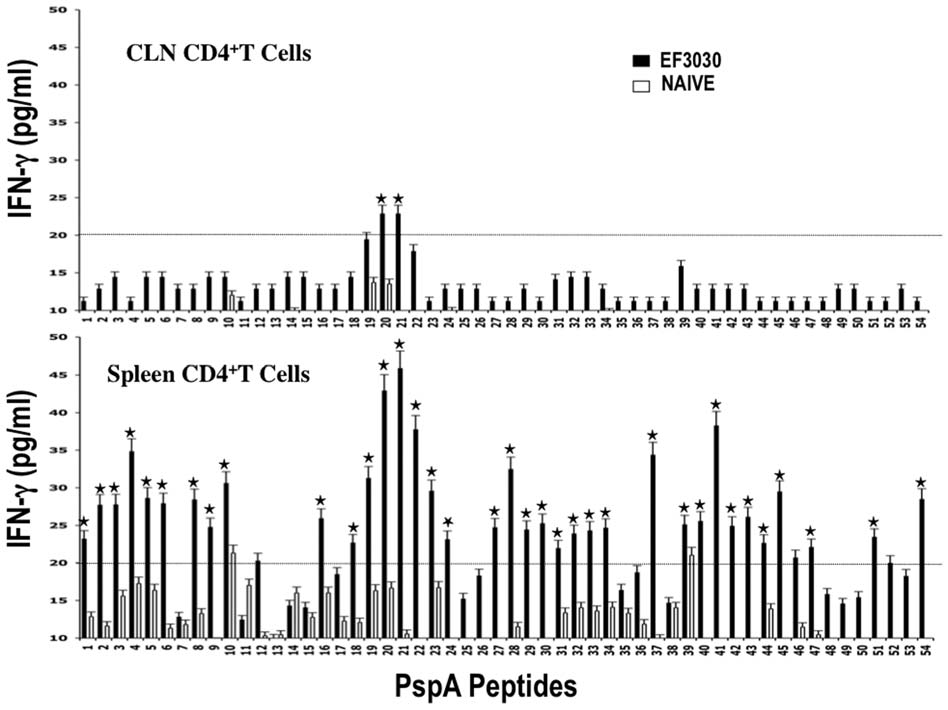
PspA peptide-specific IFN-γ secretion by CD4^+^ T cell following pneumococcal challenge. Groups of 10 F1 (B6×Balb/c) mice were intranasally challenged with 10^7^ CFUs of *S. pneumonia* strain EF3030 in a 15 µl volume of Ringer's solution. Spleen and cervical lymph node (CLN) lymphocytes were isolated from mice, 28 days after intranasal challenge with *Streptococcus pneumonia* strain EF3030 (▪) and naïve (□). CD4^+^ T cells were incubated with 1 µM of PspA peptide (15 amino acid peptides that overlapped every 11 residues) plus mitomycin C-treated naïve syngeneic feeder cells, for 3 days, at a ratio of 5∶1×10^6^ cells. The results were expressed as the mean ± the standard error mean (SEM) of IFN-γ supernatant levels from 3 replicate determinations of three independent experiments. IFN-γ production of cultured supernatants was determined by Luminex capable of detecting >2 pg/ml of IFN-γ.

**Figure 4:**
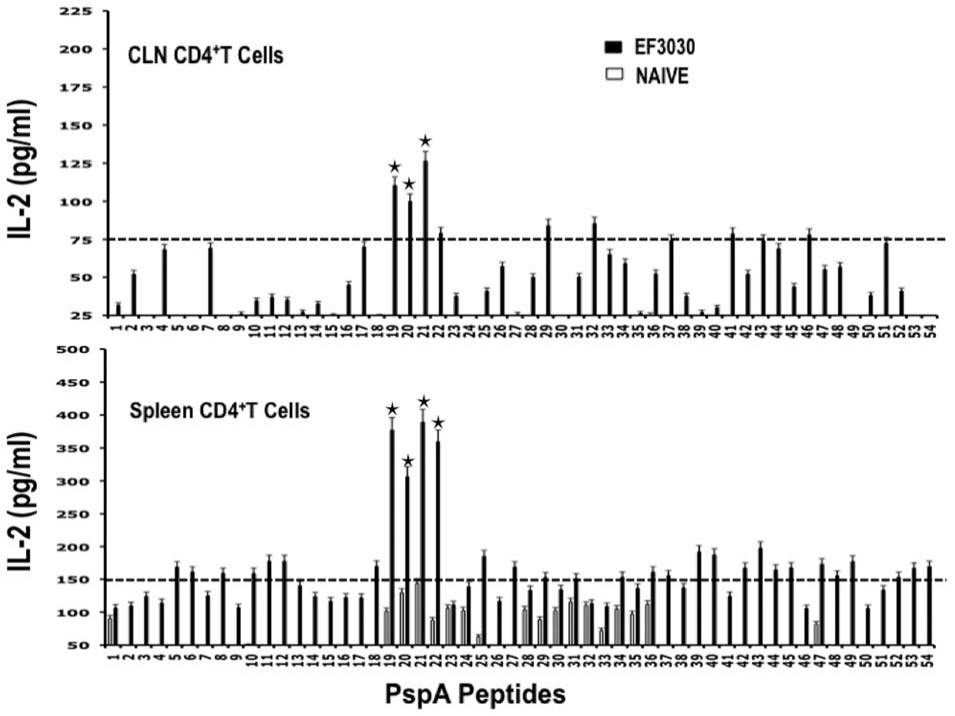
PspA peptide-specific IL-2 secretion by CD4^+^ T cell following pneumococcal challenge. Groups of 10 F1 (B6×Balb/c) mice were intranasally challenged with 10^7^CFUs of *S. pneumonia* strain EF3030 in a 15 µl volume of Ringer's solution. Spleen and Cervical lymph node (CLN) lymphocytes were isolated from mice, 28 days after intranasal challenge with *Streptococcus pneumoniae* strain EF3030 (▪) and naïve (□). CD4^+^ T cells were incubated with 1 µM of PspA peptide (15 amino acid peptides that overlapped every 11 residues) plus mitomycin C-treated naïve syngeneic feeder cells, for 3 days, at a ratio of 5∶1×10^6^ cells. The results were expressed as the mean ± the standard error mean (SEM) of IL-2 supernatant levels from 3 replicate determinations of three independent experiments. IL-2 production of cultured supernatants was determined by Luminex capable of detecting >2 pg/ml of IL-2.

In general, pneumococcal infection resulted in significantly higher HTL cytokine secretion by *ex vivo* PspA peptide-stimulated CD4^+^ T cells from the spleen as well as CLNs of *S. pneumonia* strain EF3030-challenged mice, than compared to naïve mice (Figures 3 and 4). In contrast to proliferation responses, spleen-derived CD4^+^ T cells from *S. pneumonia* strain EF3030-challenged mice secreted higher levels of IFN-γ and IL-2 after PspA peptide *ex vivo* stimulation than did similar cells from CLNs. HTLs from CLNs of pneumococcal-challenged mice, significantly responded to PspA peptides 20 and 21. CD4^+^ T cells isolated from the spleen and CLNs also significantly secreted Th2 cytokines after *ex vivo* stimulation of PspA peptides, than compared to naïve mice.

**Figure 5:**
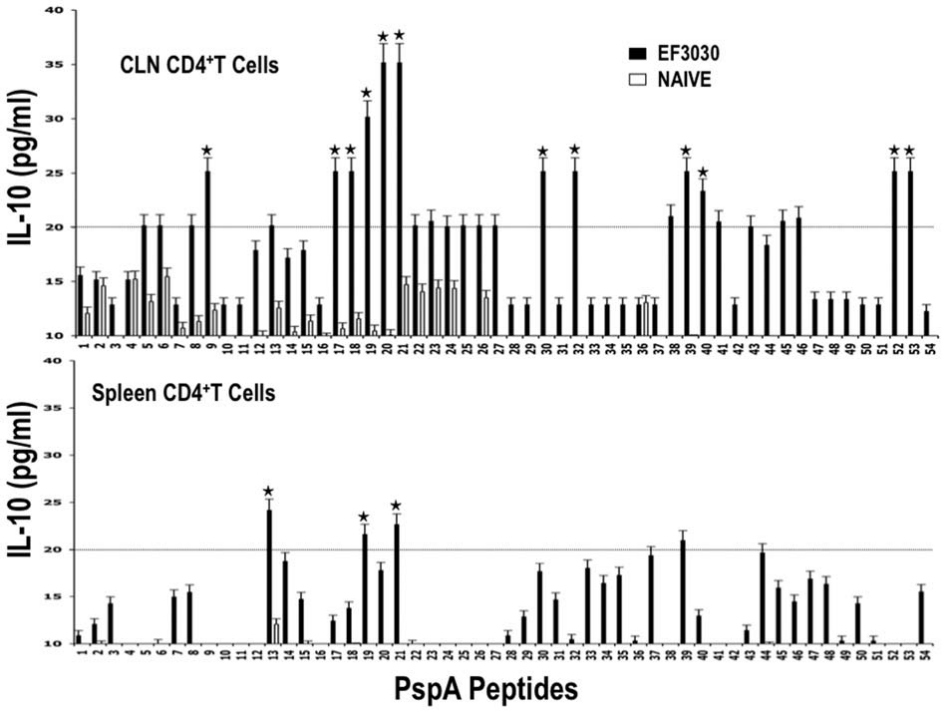
PspA peptide-specific IL-10 secretion by CD4^+^ T cell following pneumococcal challenge. Groups of 10 F1 (B6×Balb/c) mice were intranasally challenged with 10^7^CFUs of *S. pneumonia* strain EF3030 in a 15 µl volume of Ringer's solution. Spleen and Cervical lymph node (CLN) lymphocytes were isolated from mice, 28 days after intranasal challenge with *Streptococcus pneumonia* strain EF3030 (▪) and naïve (□). CD4^+^ T cells were incubated with 1 µM of PspA peptide (15 amino acid peptides that overlapped every 11 residues) plus mitomycin C-treated naïve syngeneic feeder cells, for 3 days, at a ratio of 5∶1×10^6^ cells. The results were expressed as the mean ± the standard error mean (SEM) of IL-10 supernatant levels from 3 replicate determinations of three independent experiments. IL-10 production of cultured supernatants was determined by Luminex capable of detecting >2 pg/ml of IL-10.

**Figure 6:**
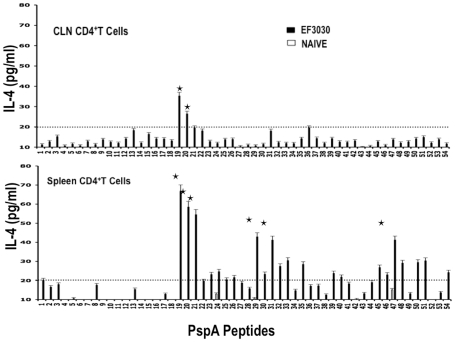
PspA peptide-specific IL-4 secretion by CD4^+^ T cell following pneumococcal challenge. Groups of 10 F1 (B6×Balb/c) mice were intranasally challenged with 10^7^CFUs of *S. pneumonia* strain EF3030 in a 15 µl volume of Ringer's solution. Spleen and Cervical lymph node (CLN) lymphocytes were isolated from mice, 28 days after intranasal challenge with *Streptococcus pneumonia* strain EF3030 (▪) and naïve (□). CD4^+^ T cells were incubated with 1 µM of PspA peptide (15 amino acid peptides that overlapped every 11 residues) plus mitomycin C-treated naïve syngeneic feeder cells, for 3 days, at a ratio of 5∶1×10^6^ cells. The results were expressed as the mean ± the standard error mean (SEM) of IL-4 supernatant levels from 3 replicate determinations of three independent experiments. IL-4 production of cultured supernatants was determined by Luminex capable of detecting >2 pg/ml of IL-4.

**Figure 7:**
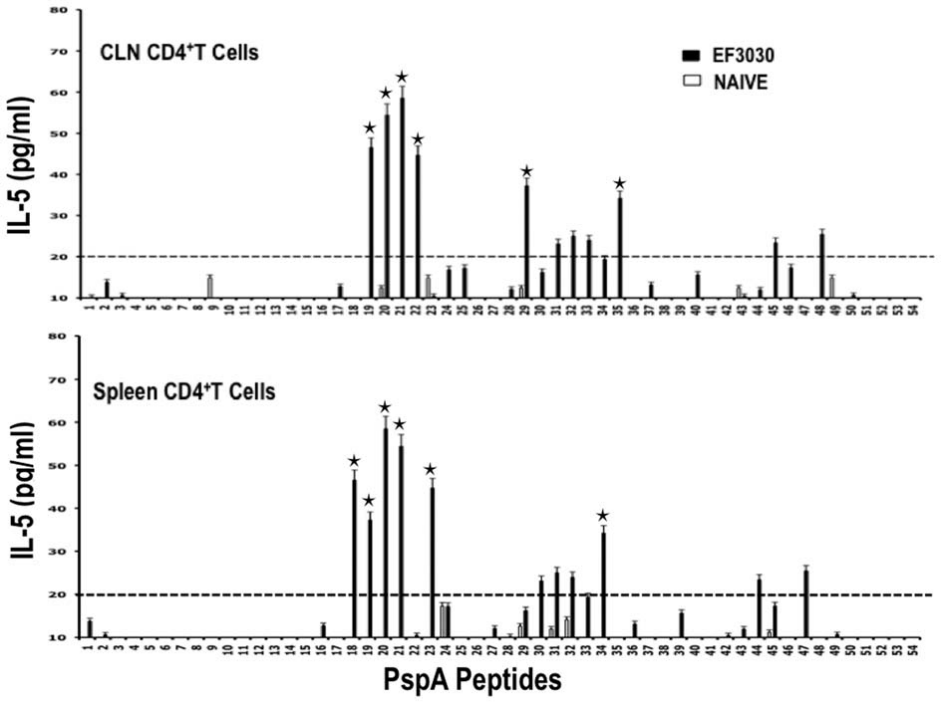
PspA peptide-specific IL-5 secretion by CD4^+^ T cell following pneumococcal challenge. Groups of 10 F1 (B6×Balb/c) mice were intranasally challenged with 10^7^CFUs of *S. pneumonia* strain EF3030 in a 15 µl volume of Ringer's solution. Spleen and Cervical lymph node (CLN) lymphocytes were isolated from mice, 28 days after intranasal challenge with *Streptococcus pneumonia* strain EF3030 (▪) and naïve (□). CD4^+^ T cells were incubated with 1 µM of PspA peptide (15 amino acid peptides that overlapped every 11 residues) plus mitomycin C-treated naïve syngeneic feeder cells, for 3 days, at a ratio of 5∶1×10^6^ cells. The results were expressed as the mean ± the standard error mean (SEM) of IL-5 supernatant levels from 3 replicate determinations of three independent experiments. IL-5 production of cultured supernatants was determined by Luminex capable of detecting >2 pg/ml of IL-5.

Similar to proliferation responses, CLN CD4^+^ T cells from *S. pneumonia* strain EF3030-challenged mice significantly secreted IL-10 following PspA peptide restimulation, with comparatively higher responses to PspA peptides 19, 20, and 21. Splenic HTLs selectively secreted significant levels of IL-10 in response to PspA peptides 13, 19, and 21 than compared to naïve mice (Figure 5). While cells from naïve mice did not significantly respond to PspA peptides, CD4^+^ T lymphocytes from *S. pneumonia* strain EF3030-challenged mice also significantly secreted IL-4 and IL-5 after PspA peptide *ex vivo* stimulation (Figures 6 and 7). In particular, there were comparatively higher responses to PspA peptides 19, 20, and 21 by splenic HTLs. Similar CLN Th2 cells secreted IL-4 in response to peptides 19 and 20 whereas heightened IL-5 secretion was noted in response to peptides 19 to 22 as well as 29 and 35, than compared to naive mice.

In summary, CD4^+^ T cells from *S. pneumonia* strain EF3030-challenged mice consistently mounted significant yet select proliferation and IL-10 responses (CLN>>spleen), IFN-γ, IL-2 and IL-4 secretion (spleen>>CLN) and IL-5 expression (spleen≤CLN) largely in response to PspA peptides 19, 20, 21, and 22. Moreover, PspA peptides 21 and 22 mounted comparatively high proliferation responses, 20 and 21 induced consistently high IFN-γ and IL-2 responses, and 19, 20, and 21 caused IL-10, IL-4 and IL-5 responses by HTLs isolated from Pneumococci-exposed mice.

### Predicted PspA Peptide-MHC Class II Alleles Binding Affinities and Correlation with Proliferation and Cytokine Secretion Responses

**Figure 8:**
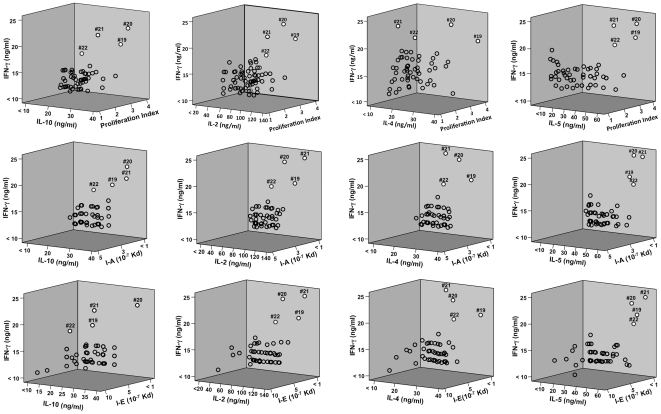
3D plot of Th1/Th2 cytokine secretion relative to proliferation or I-A/I-E predicted peptide-binding by cervical lymph node-derived CD4^+^ T cells. The panels summarize IFN-**γ**, IL-10, IL-2, IL-4, IL-5 and proliferation responses of PspA peptide-specific CD4^+^ T cells isolated from cervical lymph nodes of F1 (B6×Balb/c) mice, 28 days after *S. pneumonia* strain EF3030- challenge and predicted I-A or I-E binding affinities. Y-axis and X-axis indicate the concentration (ng/ml) of IFN-γ and IL-10, IL-2, IL-4, IL-5 respectively, secreted by PspA peptide-stimulated CD4^+^ T cells. The Z- axis represents the predicted I-A or I-E binding affinities (Kd). PspA peptides 19, 20, 21 and 22 appear as white circles, while remaining peptides are open circles.

**Figure 9:**
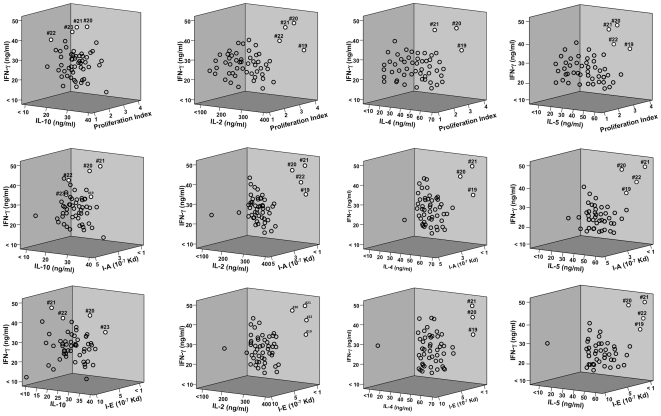
3D plot of Th1/Th2 cytokine secretion relative to proliferation or I-A/I-E predicted peptide-binding by spleen- derived CD4^+^ T cells. The panels summarize IFN-**γ**, IL-10, IL-2, IL-4, IL-5 and proliferation responses of PspA peptide-specific CD4^+^ T cells isolated from spleen of F1 (B6×Balb/c) mice, 28 days after *S. pneumonia* strain EF3030-challenge and predicted I-A or I-E binding affinities. Y-axis and X-axis indicate the concentration (ng/ml) of IFN-γ and IL-10, IL-2, IL-4,IL-5 respectively, secreted by PspA peptide-stimulated CD4^+^ T cells. The Z-axis represents the predicted I-A or I-E binding affinities (Kd). PspA peptides 19, 20, 21 and 22 appear as white circles, while remaining peptides are open circles.

PspA peptides 19, 20, 21, and 22 mounted significant HTL responses, and displayed strong predictive binding affinities to numerous HLA-DR, -DQ, and -DP as well as I-A^b^ and I-E^d^ haplotypes. This is best illustrated by viewing a 3-dimensional plot of the proliferation index as well as IFN-γ, IL-10, IL-2, IL-4, and/or IL-5 responses compared with MHC allele binding affinities (Figures 8 and 9). PspA peptide-specific T cell proliferation and IFN-γ, IL-10, IL-2, IL-4 and IL-5 secretion by CLN and splenic CD4^+^ T cells from *S. pneumonia* strain EF3030-challenged mice was higher than the naïve group. In general, CLN HTLs from mice previously challenged with *S. pneumonia* strain EF3030) secreted high levels of IFN-γ, IL-2, IL-4, IL-5 and IL-10 as well as enhanced proliferation in response to PspA peptides (19, 20>21, 22) stimulation.

PspA peptides 19, 20, 21, and 22 were predicted to bind I-A^b^/I-A^d^, I-A^b^/I-E^b^, I-A^b^ and I-A^b^/I-A^d^, respectively, with IC_50_<500 nM. From these, PspA peptide 20 was predicted to have marginal binding affinities to I-A^b^ and I-E^b^ with IC_50_ = 485 and 493 nM, respectively. This also corresponded with relatively high IL-10 responsiveness. Spleen-derived CD4^+^ T cells secreted significant amounts of IFN-γ, IL-2, IL-4 and IL-5 as well as proliferated in response to PspA peptides 19, 20, 21, and 22 (i.e., PspA_199–246_) stimulation from mice previously challenged with *S. pneumonia* strain EF3030. Peptide 20 or 23 stimulation of splenic HTLs resulted in comparatively high secretion of IL-10. Similar to PspA peptide 20, peptide 23 was predicted to have moderate I-A^b^ and I-E^b^ binding affinity i.e., IC_50_ = 452 and 412 nM, respectively. Peptides that induced spleen-derived CD4^+^ T cells to secrete high levels of Th1 (IFN-γ/IL-2) and Th2 (IL-4/IL-5) cytokines also correlated with relatively high MHC binding affinities. It is important to note that several PspA peptides predicted to tightly bind I-A and/or I-E alleles did not always correspond with elevated cytokine secretion (e.g., peptides 6, 18, 30, and 53).

## Discussion

The immune system is remarkably robust in responding to a multitude of foreign antigens. T cells are crucial for generating an efficient immune response following recognition of foreign antigen in the context of MHC. The polymorphism of MHC genes leads to differences in immune responsiveness. While peptide vaccines potentially circumvent the problem of using whole antigen or attenuated pathogens as vaccines, this approach is impeded by the exhaustive MHC repertoire [24]. Hence, the identification of optimal or common HTL epitopes is imperative in mounting a protective immune response. To this end, MHC α chains have limited variability compared to MHC β chains suggesting that the binding affinity of MHC β chains dictates antigenic specificity [25], [26]. This restricts the utility of peptides as vaccines. The discovery of “promiscuous” or “universal” peptides that can bind multiple HLA (β chain) allele would solve many of these problems. While HLA-transgenic mice have been used to map HTL epitopes [27], the limited number of HLA transgenic mice are not representative of all populations. Hence, the current study is the first of many to map clinically relevant HTL pneumococcal epitopes. We have utilized *in silico* methods for predicting class II-restricted peptides and evaluated immunogenicity by *ex vivo* peptide-restimulation.

Protein secondary structure consists of regular elements such as α-helices and β-sheets, and irregular elements such as β-bulges, random coils, and tight turns. Tight turns are generally classified as δ-, γ-, β-, α-, and π-turns according to the number of residues involved [28]. β-turns have important biological tasks [29]. We predicted β-turns in PspA using a new and highly accurate secondary structure prediction software, PSIPRED, which incorporates two feed forward neural networks that perform an analyses on PSI-BLAST position-specific-iterated- BLAST peptide sequence [30]. β-turns were abundant in PspA Region C, which did not have immunodominant HTL epitopes. While β-sheet structures were not detected, analysis revealed PspA has α-helical secondary structure content and is predominantly a coiled-coil structure. These structural properties correlate with PspA function and anti-complement activity [31]. In general, PspA peptides with continuous helix or strand predicted secondary structures were not considered immunodominant; instead, PspA peptides 19, 20, 21, and 22 (or PspA_199–246_) were estimated to predominantely have a coiled secondary structure.

In addition to protein secondary structure, proteases and MHC class II co-mingle in the antigen-processing compartment and compete for peptides that satisfy requirements for protease or MHC recognition, respectively. Indeed, several proteases are implicated in processing antigen and the MHC class II-bound invariant chain [32], [33], [34]. The proteolytic separation of MHC class II-bound epitopes was found to be a rate-limiting step in the presentation of T cell epitopes [35]. The level and activity of N endopeptidases can directly control the proteolysis and presentation of T cell epitopes [36]. In contrast to other proteases, N endopeptidase is required for both antigen and invariant chain (Ii) processing [37], [38], [39], [40]. Hence, N endopeptidase can have both positive and negative effects on the outcome of antigen processing [23], [41], [42]. Future studies will be required to verify whether the candidate HTL peptides are able to induce protective immunity against to pneumococcal infection.

PspA is highly immunogenic and is considered a promising vaccine candidate for combating pneumococcal infection [43], [44], [45]. In our model, *S. pneumonia* strain EF3030 promoted substantial PspA peptide-specific HTL responses. We show that PspA_199–246_ (i.e., PspA peptides 19, 20, 21, and 22) is highly immunogenic and likely encompasses HLA class-binding epitopes to support pneumococcal immunity. Further, PspA _199–246_ is highly conserved among 100 different family 1 *S. pneumonia* strains (Table 3). In confirmation, Region B lies within PspA_199–246_ and was found to be important in eliciting protective pneumococcal immunity [46]. Taken together, our findings support the rationale for additional studies to explore the utility of PspA_199–246_-based vaccines.

*S. pneumonia* has co-evolved with man and no doubt has numerous immune evasion mechanisms to avoid detection by T cells. From the pathogen's perspective, it would be critical to maintain PspA function, while reducing detection of a T cell immuno-dominant epitope (i.e., peptide 21). PspA peptide 21 restimulation of pneumococcal-infected mice induced significant cytokine production and proliferation, yet was predicted to be poorly recognized by mouse and human MHC class II alleles. In contrast, other immunodominant regions exist within peptides 38 to 41 and might be protective since they invoked CD4^+^ T cell proliferation as well as T helper cytokine responses. However, these peptides reside in Region C, which has several potential, N endopeptidase sites along with β turn secondary structures that would optimally expose these sites for cleavage. In particular, peptide 40 has a highly conserved N endopeptidase site (i.e., NxN) that lies in the middle of a pronounced β turn secondary structure (ttttt). While this intact peptide would potentially bind several MHC class II alleles, it is also likely that it would be cleaved before or after MHC-binding by N endopeptidases.

The Th1-associated cytokine, IL-2 promotes T cell proliferation. Our data show PspA_199–246_ peptides mounted comparatively high IL-2 and proliferation HTL recall responses in mice previously challenged with *S. pneumonia* strain EF3030. Another Th1 cytokine, IFN-γ, is required for protective pneumococcal immunity [47]. CD4^+^ T cells from *S. pneumonia* strain EF3030-challenged mice secreted significant amounts of IFN-γ following *ex vivo* PspA peptide re-stimulation. IFN-γ blockade accelerated the death of animals during pneumococcal infection [48], whereas treatment of mice with IFN-γ enhanced the survival of mice [49]. However, confounding studies suggest that too much IFN-γ and too little IL-10 can inhibit pneumococcal clearance during *S. pneumonia* infection that is secondary to influenza virus infection [50].

IL-10 has been suggested to be both deleterious and important for pneumococcal immunity. On one hand, administration of anti-IL-10 antibody was shown to enhance pneumococcal immunity [51], while others showed this Th2-associated cytokine is critical for MARCO-1 expression and subsequent pneumococcal clearance [50]. We show that PspA_199–246_ stimulates pneumococcal strain EF3030-primed CD4^+^ T cells to secrete IL-10. Interestingly, HTLs from CLN mounted IL-10 responses to more peptides, than similar cells isolated from the spleen. Perhaps this contributes to establishing pneumococcal carriage by supporting selective pneumococcal clearance by CLN>>spleen antigen-presenting cells after stimulation with CD4^+^ T cell-derived IL-10, whereas IFN-γ-secreting HTLs might support spleen>>CLN macrophages activation and/or internalization of *S. pneumonia*.

In the absence of IL-10, a marked increase in pro-inflammatory cytokines is induced during pneumococcal infection [52]. To this end, IL-10 plays an indispensable role in mucous cell metaplasia and hyperplasia. IL-10 attenuates the proinflammatory cytokine response and its absence hampers effective clearance of the infection, and reduces survival of pneumococcal infection [53]. We have shown that CCL5 inhibition resulted in lower IFN-γ-secreting CD4^+^ T cells and significantly more PspA-specific IL-10-producing CD4^+^ T cells, which corresponded with the transition from pneumococcal carriage to lethal pneumonia [45], [54]. Thus, the precise contribution of IL-10 in pneumococcal immunity remains uncertain, but the preponderance of the evidence suggests excessive IL-10 responses play a deleterious role in pneumococcal immunity, but moderate levels of this cytokine are required for optimal adaptive (humoral) immune responses to *S. pneumonia* and reduced mucosal hyperplasia.

An effective intranasal conjugate pneumococcal vaccine using interleukin-12 (IL-12) as a mucosal adjuvant induced protection and increased expression of lung and splenic IFN-γ and IL-10 mRNAs and protected mice from lethal challenge [55]. Thus, interplay and requirement of the HTL-derived IFN-γ and IL-10 in pneumococcal carriage and pneumonia will require further study. In addition, the adjuvants or cytokines, e.g., IL-12, required by antigen presenting cells to promote IFN-γ and IL-10 secreting, PspA-specific T cells will be addressed in the future.

Some studies suggest that Th2 cytokines do not support optimal pneumococcal immunity. Mice primed to mount Th2 cell responses followed by pneumococcal infection showed an increase in the number of Pneumococci and an increase in sinus inflammation than compared to naive or Th1 -primed groups [56]. IL-4 plays a central role in directing the development of the Th2 phenotype and IL-4 responses in lung have been associated with an increased risk to pneumococcal infection [57]. While IL-4 does not stimulate T cell proliferation, it induces the growth of lymphoblasts [58]. IL-5 was originally defined as a Th2 cell-derived cytokine that triggers B cell activation and differentiation into plasma cells [59]. PspA_199–249_-specific HTLs from *S. pneumonia* strain EF3030-challenged mice secreted significant amounts of IL-4 (spleen>>CLN) and IL-5 (spleen≤CLN) largely in response to PspA peptides 19, 20, 21, and 22. However, the uncertain role of IL-4 and IL-5 in pneumococcal cellular immunity makes correlations of these cytokines with protective immunity difficult.

The role of Th17 cells in pneumococcal immunity has not been extensively studied. However, recent reports suggest that IL-17A supports antibody responses to pneumococcal capsular polysaccharides [60]. Mice lacking the IL-17A receptor or mice with neutrophil depletion are more susceptible to pneumococci [61]. Additional studies on the role of HTL-derived IL-17 would greatly contribute to the field and will be required to understand how secretion of this cytokine correlates with pneumococcal immunity.

While the precise role of peptide MHC class II interactions that determine protective pneumococcal immunity are not known, this study addresses important questions that are relevant to MHC polymorphisms and antigen responsiveness. A number of studies have definitively proven a cause and effect relationship between human MHC genes and resistance to infection [62], [63] as well as autoimmune diseases [64]. I-A, which is highly homologous to HLA-DQ [65], typically restricts antigen-specific CD4^+^ T cells in mice, whereas I-E (homologous to HLA-DR) [66], [67], [68] has been reported to control non-responsiveness through antigen-specific suppressor cells [69]. Further studies will be required to determine whether I-E or I-A as well as DQ or DR molecules might be involved in pneumococcal antigen non-responsiveness or cytokine secretion in mouse or man, respectively. To this end, many of the PspA peptides were predicted to bind I-A, while relatively few were predicted to bind I-E. These studies support the use of *in silico* and *in vivo* methods to validate T cell responsiveness to PspA peptide-based vaccines.

## Materials and Methods

### Animals

Female F1 (B6×Balb/c) mice, aged 8 to 12 weeks, contain MHC class II haplotype and corresponding TCR diversity that approaches those seen in man [70], [71] and were purchased from Jackson Laboratories. All mice were housed in horizontal laminar flow cabinets free of microbial pathogens. Routine antibody screening for a large panel of pathogens and routine histological analysis of organs and tissues were performed to insure that mice were pathogen free.

### *S. pneumonia* Strain EF3030 Growth and Challenge

*S. pneumonia* capsular strain EF3030 was among the human isolates of capsular group 19 that were previously examined and found to be relatively non-invasive in mice [72]. Pneumococci were grown in Todd Hewitt broth and stored frozen in aliquots at −80°C, in 20% glycerol, in sterile lactated Ringer's solution (Ringer's) (Abbott Labs, North Chicago, IL) [73], [74]. To establish nasal carriage, Pneumococci were introduced into groups of mice (8 to 12 week old) by nasal administration. The animals were anesthetized with ketamine (100 mg/ml) and xylazine (20 mg/ml), mixed at a 4∶1 (vol/vol) ratio. The anesthesia mixture was injected intramuscularly into the right hamstring muscle at a dose of 100 mg of ketamine per kg of body weight. After anesthesia was established, the mice were inoculated with approximately 10^7^ colony forming units (CFU) of *S. pneumonia* strain EF3030 in 15 µl of Ringer's solution using a 25-gauge ball-tipped gavage needle [75]. Experimental groups consisted of 10 mice and studies were repeated 3 times. The guidelines proposed by the committee for the Care of Laboratory Animal Resources Commission of Life Sciences - National Research Council were followed to minimize animal pain and distress. All procedures involving mice were approved by the Morehouse School of Medicine Committees (IACUC).

### Pneumococcal Antigens

54 overlapping peptides, spanning the entire length of *S. pneumonia* strain D39/R6 PspA protein sequence (NCBI Accession # NP_357715), starting with the first 15 residues at the N- terminus, was synthesized by the multipin synthesis method by Chiron Mimotopes Peptide Systems. Peptides overlapped by four amino acids (Table 1) and were acetylated at the N- terminus and ended with a COOH-terminal. Purity of these peptides was approximately 95%. The peptides were dissolved in a mixture (v/v) of 75% dimethyl sulfoxide and 25% water, to a concentration of 70 mM, divided into small aliquots and stored frozen at −80°C.

### Tissue Collection and Cell Isolation

Mice were sacrificed by CO_2_ inhalation to collect spleen and CLNs for single cell isolation of lymphocytes 28 days following *S. pneumonia* strain EF3030 challenge. Individual single cell suspensions of spleen and CLNs were collected and prepared by aseptically removing tissues and passage through a sterile wire screen. Unpooled CD4^+^ T cells were further separated by OctoMACS™ (Miltenyi Biotec) using negative selection. Remaining (non-CD4^+^) cells, were used as accessory feeder cells for antigen peptide-specific stimulation assays after mitomycin C (Sigma-Aldrich) treatment.

### Cytokine Quantitation by Luminex™ Analysis

Purified CD4^+^ T cells and mitomycin C-treated feeder cells were cultured at a density of 5×10^6^ and 10^6^ cells per ml, respectively, in complete medium containing 1 µM of each PspA peptide at 37°C in 5% CO_2_. For the assessment of cytokine production, 100 µL of culture supernatants from 96-well flat bottom plates (Corning Glass Works) were harvested 3 days after *ex vivo* PspA peptide stimulation to determine the levels of IL-10 and IFN-γ secreted by CD4^+^ T cells. phorbol-12-myristate-13-acetate (PMA) 1 µg/ml was used as a positive control , ovalbumin (1 µg/ml) and medium only is used as negative control to reduce the background reading. Supernatant cytokine levels were determined by the Beadlyte™ mouse multi-cytokine detection (Bio-Rad). Briefly, filter bottom ELISA plates were rinsed with 100 µL of Bio-plex assay buffer and liquid was removed using a Millipore™ Multiscreen Separation Vacuum Manifold System set at 5 mm Hg. Analyte beads in assay buffer were added to the wells followed by 50 µL of serum or standard solution. The plates were incubated for 30 minutes at room temperature with continuous shaking (at setting #3) using a Lab-Line™ Instrument Titer Plate Shaker. The filter bottom plates were washed, as before, and centrifuged at 300×g for 30 seconds. Subsequently, 50 µL of anti-mouse IL-10 or IFN-γ antibody-biotin reporter solution was added in each well, after which the plates were incubated with continuous shaking for 30 min followed by centrifugation and washing. Next, 50 µL streptavidin-phycoerythrin (PE) solution was added, and the plates were incubated with continuous shaking for 10 min at RT. 125 µL of Bio-plex assay buffer was added, and Beadlyte™ readings were measured using a Luminex™ System and calculated using Bio-plex™ software (Bio-Rad). The cytokine Beadlyte™ assays were capable of detecting >5 pg/mL for each analyte.

### Cell Proliferation

Lymphocyte proliferation was measured by a 5-Bromo-2′-deoxy uridine (BrdU) absorption and detection (Roche Diagnostics). In brief, purified CD4^+^ T cells were cultured at a density of 5×10^6^ cells/mL, with 10^6^ mitomycin C-treated feeder cells/mL in complete medium containing 1 µM of PspA peptide at 37°C in 5% CO_2_. After 2 days of *ex vivo* antigen stimulation, cells were transferred to polystyrene 96 well plates (Corning Glass Work). 10 µL of BrdU labeling solution (10 µM final concentration per well) were added and incubated for 18 hours at 37°C with 5% CO_2_. The cells were then fixed and incubated with 100 µL of nuclease in each well for 30 minute at 37°C. The cells were washed with complete media and incubated with BrdU-POD solution for 30 minute at 37°C. BrdU incorporation was developed with an 2,2′–azino-bis 3- ethylbenzthiazoline-6-sulfonic acid (ABTS) solution and optical density (OD) was read at 450 nm. The proliferation index (PI) was calculated as follows. Antigen-specific CD4^+^ T cell proliferation was obtained by measuring 5-Bromo-2′-deoxy uridine (BrdU) incorporation, according to manufacturer's instructions (Roche Diagnostics). BrdU absorption or optical density at 450 nm (OD_450_) was detected using a scanning multi-well SpectraMax 250 spectrophotometer (Molecular Devices). PI = OD_450_ in peptide stimulated cell/OD_450_ in un-stimulated cells ×100. The results were expressed as mean ± the standard error mean (SEM) of the response of 3 replicate determinations from three independent experiments. Statistical significance was assessed by student's t test.

### MHC Class II Epitope Prediction Using External Tools

IEDB (http://www.immuneepitope.org/), SYFPEITHI (http://www.syfpeithi.de/), SVMHC (http://www.bs.informatik.unituebingen.de/SVMHC/), RANKPEP (http://bio.dfci.harvard.edu/RANKPEP/), and MHCPred (http://www.jenner.ac.uk/MHCPred) external software(s) were used to predict peptide binding affinities to mouse I-A and I-E as well as HLA-DR, -DP and -DQ. In brief, for average relative binding (ARB) evaluation, 10-fold cross validation results stored at IEDB were used to estimate performance. Because the binding of peptides to MHC class II molecules is not dependent on exact size, derivation of MHC class II ARB matrices followed an iterative procedure. For the first iterative step, a matrix was generated from a set of nine-residue core sequences randomly obtained from each peptide sequence in the training set. For subsequent cycles, nine-residue core sequences were used to generate a matrix. The overall binding affinity of a peptide was predicted using the highest scoring nine-residue core sequence. For the SYFPEITHI prediction, we patched each testing peptide with three glycine residues at both ends before evaluation for prediction. This was recommended by the creators of SYFPEITHI method to ensure that all potential binders were correctly presented to the prediction algorithm. For all other methods, the original tested peptides were submitted directly for prediction. Peptide sequences were sent to web servers one at a time and predictions were extracted from the server's response. To assign a single prediction for peptides longer than nine amino acids in the context of tools predicting the affinity of 9 core-binding regions, we took the highest affinity prediction of all possible 9-mers within the longer peptide as the prediction result. For each MHC class II molecule whose binding can be predicted by three or more algorithms, the top three methods were selected that gave the best performance. For each method, peptides were tested and ranked by their scores with higher ranks for better binders. For each tested peptide, three ranks from different methods were taken and the median rank was taken as the consensus score. Peptides were classified into binders (IC_50_<500 nM) and nonbinders (IC_50_≥500 nM), as practical cutoffs.

### Statistics

Data are expressed as the mean ± SEM and compared using a two-tailed student's *t*-test or an unpaired Mann Whitney U test. The results were analyzed using Microsoft Excel for Macintosh computers and were considered statistically significant if *p* values were less than 0.01. When cytokine or antibody levels were below the detection limit (BD), they were recorded as one-half the lower detection limit for statistical analysis.
